# Surf and turf: A dataset of stable isotope values of plants and animals from southern California

**DOI:** 10.1016/j.dib.2021.107380

**Published:** 2021-09-20

**Authors:** Mikael Fauvelle, Andrew D. Somerville

**Affiliations:** a Department of Archaeology and Ancient History, Lund University, Helgonavägen 3, Box 192, Lund 221 00, Sweden; b Department of World Languages and Cultures, Iowa State University, 505 Morrill Rd., Ames, IA 50011, USA

**Keywords:** Paleodiet, Stable isotopes, California, North America, Historical ecology

## Abstract

This article presents baseline isotope values for plant and animal samples collected from across southern California. A total of 80 samples representing 50 species were collected and analyzed for this project. This original dataset includes 31 plant species, 13 finfish species, 3 mollusk species, 2 crustacean species, and 1 echinoderm. Plant samples were collected by the authors and an undergraduate research assistant in San Diego and Santa Barbara counties. Animal samples were procured from local fishers in San Diego and Santa Barbara. All samples were subjected to stable isotope analysis at the University of California, San Diego (UCSD). In this paper we present our new δ^13^C and δ^15^N data alongside previously published values from three other studies [1], [2], [3]. The previously published values reproduced here were derived from a mix of modern and archaeological samples. Together, these data represent the most complete dataset currently available for southern California baseline stable isotope values. The full combined dataset can be found in the Supplemental Data included with this paper, while Table 1 presents a comparison of defatted and untreated faunal specimens. These data will be of use to archaeologists and ecologists conducting future isotope studies on the Pacific Coast of North America.

**Specifications Table**

**Table:** 

Subject	Archaeology
Specific subject area	Historical ecology and paleodiets in southern California.
Type of data	Table
How data were acquired	Plant and animal samples were collected in San Diego and Santa Barbara counties of southern California and processed at the UCSD Paleodiet Laboratory, directed by Dr. Margaret Schoeninger. Plant samples were cleaned, shredded, dried, then reduced to a fine powder using an agate mortar and pestle and a fine sieve (U.S. 100, 149 microns). Finfish and invertebrate soft tissue samples were removed from the organism with a stainless-steel scalpel and cut into small pieces (∼2, 3 mm diam.). Animal samples were frozen and then lyophilized using a Labconco benchtop freeze dryer. Stable isotope analyses for δ^13^C and δ^15^N values for all samples were accomplished by combusting samples within tin capsules in a Costech 4010 Elemental Analyzer coupled to the Thermo-Finnigan Delta XP Plus mass spectrometer.
Data format	Raw
Parameters for data collection	We targeted the collection of plant and animal samples on species that were mentioned in ethnohistoric literature as being important foods to Native Americans of southern California. Marine foods in particular were targeted due to a lack of previous coverage.
Description of data collection	Plant samples were collected by the authors or an undergraduate research assistant from undeveloped areas in Santa Barbara and San Diego counties. Animal samples were procured directly from local fishers in San Diego and Santa Barbara.
Data source location	City/Town/Region: Santa Barbara County Country: USA City/Town/Region: San Diego County Country: USA Primary data sources: [1] Goldberg (1993) [2] Newsome et al. (2010) [3] Somerville et al. (2018)
Data accessibility	With the article
Related research article	[4] Fauvelle and Somerville (2021)

**Value of the Data**

• These data present the most complete set of δ^13^C and δ^15^N values currently available for plant and animal food sources that may have been used by ancient populations in southern California.
• These data will be of interest to Archaeologists and Historical Ecologists working in southern California or the Pacific Coast of North America.
• Future studies of paleodiets in southern California or Pacific North America will be able to use the values presented here as a baseline to interpret human isotope data.
• Dataset presented here greatly expands coverage of marine and maritime foods compared to previous baseline stable isotope studies.
• Studies of historical ecology or human paleodiets focusing on marine and coastal environments around the world will be able use these values as a point of comparison.

## 1 Data Description

Supplemental Data. This table presents a combined set of available baseline stable isotope data for possible human foods in southern California. This table includes values from 80 samples processed by the authors for this study, as well as 79 values taken from previous studies. Together these data represent the largest currently available baseline stable isotope library for southern California.

Table 1. This table presents a comparison of stable isotope values for defatted and untreated faunal specimens processed for this study (see experimental design).

**Table 1:** Comparison of defatted and untreated faunal specimens.

Lab Number	Pair	Common Name	Scientific Name	Treated for Lipids	Location	%N	%C	δ^15^N	δ^13^C	C:N	Δ^13^C	Δ^15^N
AS-0882	1	Rock crab	Cancer productus/C. antennarius	No	San Diego	13.2	44.1	15.7	-15.8	3.9		
AS-0883	1	Rock crab	Cancer productus/C. antennarius	Yes	San Diego	15.1	50	15.7	-15.9	3.9	0	0
AS-0884	2	Abalone	Haliotis	No	San Diego	9.9	42	7.6	-12.9	5		
AS-0885	2	Abalone	Haliotis	Yes	San Diego	13.6	49.2	9	-11.7	4.2	-1.4	-1.2
AS-0888	4	Snapper	Lutjanus	No	San Diego	6.5	53.3	14.5	-19.6	9.6		
AS-0889	4	Snapper	Lutjanus	Yes	San Diego	8.1	52.1	14.9	-18.2	7.5	-0.4	-1.4
AS-0890	5	Snapper	Lutjanus	No	San Diego	15	47.1	16.1	-15.8	3.7		
AS-0891	5	Snapper	Lutjanus	Yes	San Diego	15.6	49.9	16.7	-16	3.7	-0.6	0.2
AS-0925	8	Opah	Lampris guttatus	No	San Diego	14.4	46.4	13.7	-18.7	3.8		
AS-0924	8	Opah	Lampris guttatus	Yes	San Diego	16	52.3	13.9	-18.5	3.8	-0.2	-0.1
AS-0926	10	Opah	Lampris guttatus	No	San Diego	13.6	50.4	10.7	-20	4.3		
AS-0927	10	Opah	Lampris guttatus	Yes	San Diego	15.1	54.3	11	-19.6	4.2	-0.3	-0.4
AS-0928	11	Silk Snapper	Lutjanus peru	No	San Diego	15.1	48.3	17.2	-17.3	3.7		
AS-0929	11	Silk Snapper	Lutjanus peru	Yes	San Diego	15.3	50.6	18.2	-17.3	3.8	-1	0
AS-0930	12	Silk Snapper	Lutjanus peru	No	San Diego	14.4	47.7	17.2	-18.1	3.9		
AS-0931	12	Silk Snapper	Lutjanus peru	Yes	San Diego	16.2	51.3	17.9	-17.6	3.7	-0.7	-0.5
AS-0932	13	Silk Snapper	Lutjanus peru	No	San Diego	15.5	49.7	17.7	-17.4	3.7		
AS-0933	13	Silk Snapper	Lutjanus peru	Yes	San Diego	15.6	51.3	18.3	-17.4	3.8	-0.6	0
AS-0934	14	Mako	Isurus oxyrinchus	No	San Diego	16.8	47.7	17	-17.7	3.3		
AS-0935	14	Mako	Isurus oxyrinchus	Yes	San Diego	16.4	49.5	17.6	-17.2	3.5	-0.6	-0.5
AS-0936	15	Mako	Isurus oxyrinchus	No	San Diego	15.1	48.4	17.6	-17.2	3.7		
AS-0937	15	Mako	Isurus oxyrinchus	Yes	San Diego	10.8	34.9	18.2	-17.2	3.8	-0.6	0.1
AS-0938	16	Lobster	Panulirus interruptus	No	San Diego	14.3	48	15.1	-14.9	3.9		
AS-0940	16	Lobster	Panulirus interruptus	Yes	San Diego	15.5	48.9	13.7	-16.8	3.7	1.4	1.9
AS-0941	18	Lobster	Panulirus interruptus	No	San Diego	15.7	51.9	15.5	-14.9	3.8		
AS-0942	18	Lobster	Panulirus interruptus	Yes	San Diego	13.7	45.8	15.3	-16.9	3.9	0.1	2
AS-0943	20	Swordfish	Xiphias gladius	No	San Diego	14.8	48.7	16.2	-16.6	3.8		
AS-0944	20	Swordfish	Xiphias gladius	Yes	San Diego	13.4	51	14.4	-18.9	4.4	1.8	2.3
AS-0945	21	Swordfish	Xiphias gladius	No	San Diego	13.3	54.6	14.3	-18.8	4.8		
AS-0946	21	Swordfish	Xiphias gladius	Yes	San Diego	13.2	50.4	15	-18.4	4.5	-0.7	-0.4
AS-0947	22	Swordfish	Xiphias gladius	No	San Diego	13.8	54.4	15	-18.7	4.6		
AS-0948	22	Swordfish	Xiphias gladius	Yes	San Diego	14.5	48	15.7	-17.7	3.9	-0.7	-1
AS-0950	24	Sea Urchin	Strongylocentrotus purpuratus	No	San Diego	8.3	52	10	-18	7.3		
AS-0951	24	Sea Urchin	Strongylocentrotus purpuratus	Yes	San Diego	10.1	53.1	10.1	-17.7	6.1	-0.1	-0.4
AS-0952	25	Sea Urchin	Strongylocentrotus purpuratus	No	San Diego	8	52.1	10.1	-18.4	7.6		
AS-0953	25	Sea Urchin	Strongylocentrotus purpuratus	Yes	San Diego	10.1	49.4	10.5	-17.1	5.7	-0.4	-1.4
AS-0954	26	Sea Urchin	Strongylocentrotus purpuratus	No	San Diego	7.5	55.5	9.9	-18.4	8.7		
AS-0955	26	Sea Urchin	Strongylocentrotus purpuratus	Yes	San Diego	10.5	51.1	10.1	-17	5.7	-0.2	-1.5
AS-0956	27	Sea Urchin	Strongylocentrotus purpuratus	No	San Diego	6.7	54.8	9.3	-21.1	9.5		
AS-0957	27	Sea Urchin	Strongylocentrotus purpuratus	Yes	San Diego	6.7	58.7	9.5	-21.1	10.3	-0.2	0
AS-0958	28	Sea Urchin	Strongylocentrotus purpuratus	No	San Diego	7.9	54.4	10.2	-17.9	8.1		
AS-0959	28	Sea Urchin	Strongylocentrotus purpuratus	Yes	San Diego	11.5	47.7	10.8	-15.9	4.9	-0.5	-2
AS-0960	29	Skip Jack	Katsuwonus pelamis	No	San Diego	13.1	54.6	11.8	-19.9	4.9		
AS-0961	29	Skip Jack	Katsuwonus pelamis	Yes	San Diego	14.5	52.1	12.6	-18.7	4.2	-0.8	-1.2
AS-0962	30	Big Eye	Thunnus obesus	No	San Diego	15.2	48	10.7	-17.9	3.7		
AS-0963	30	Big Eye	Thunnus obesus	Yes	San Diego	15.9	52.1	11.4	-18.1	3.8	-0.7	0.2
AS-0964	31	White Sea Bass	Atractoscion nobilis	No	San Diego	13.1	42.6	18.2	-17.6	3.8		
AS-0965	31	White Sea Bass	Atractoscion nobilis	Yes	San Diego	15.8	54.5	18.9	-17.9	4	-0.7	0.3
AS-0966	32	California Skate	Raja inornata	No	San Diego	16.1	46.3	16.3	-16.7	3.3		
AS-0967	32	California Skate	Raja inornata	Yes	San Diego	16	50.1	16.7	-16.5	3.7	-0.4	-0.2
AS-0968	33	Ahi	Thunnus spc.	No	San Diego	16.1	49.3	11.4	-17	3.6		
AS-0969	33	Ahi	Thunnus spc.	Yes	San Diego	16.7	53.7	12.3	-17	3.7	-0.9	0.1
AS-0970	34	Turban Snail	Megastraea undosa	No	Santa Barbara	12.2	38.3	11.7	-20	3.6		
AS-0971	34	Turban Snail	Megastraea undosa	Yes	Santa Barbara	13.4	40.2	11.8	-19.6	3.5	-0.1	-0.4
AS-0972	35	Ono	Acanthocybium solandri	No	San Diego	15.5	48.7	14	-16.7	3.7		
AS-0973	35	Ono	Acanthocybium solandri	Yes	San Diego	16.3	51.1	14.4	-16.5	3.7	-0.3	-0.2
AS-0976	37	Yellow Tail	Seriola lalandi	No	Santa Barbara	15.2	50.3	17.7	-17.1	3.9		
AS-0977	37	Yellow Tail	Seriola lalandi	Yes	Santa Barbara	16.3	53.1	18.7	-16.8	3.8	-0.9	-0.3
AS-0978	38	Mussle	Mytilus californianus	No	Santa Barbara	12.6	45	8.8	-18.8	4.2		
AS-0979	38	Mussle	Mytilus californianus	Yes	Santa Barbara	27.4	90	11.9	-16.9	3.8	-3	-1.9
AS-0980	39	Mussle	Mytilus californianus	No	Santa Barbara	10.1	39.5	10.2	-18	4.6		
AS-0981	39	Mussle	Mytilus californianus	Yes	Santa Barbara	12	41.8	10.6	-17.3	4	-0.4	-0.7
AS-0982	40	Yellowfin	Thunnus albacares	No	San Diego	15.2	44.2	8.2	-16.8	3.4		
AS-0983	40	Yellowfin	Thunnus albacares	Yes	San Diego	17	54.4	9.8	-17.2	3.7	-1.6	0.3
AS-0984	41	Sardine	Sardinops sagax	No	San Diego	12.7	46.9	13.7	-19.2	4.3		
AS-0985	41	Sardine	Sardinops sagax	Yes	San Diego	14.2	55.2	14.3	-19.2	4.5	-0.6	0
AS-0986	42	Mussle	Mytilus californianus	No	Santa Barbara	7	25.3	8.7	-18.4	4.2		
AS-0987	42	Mussle	Mytilus californianus	Yes	Santa Barbara	9.3	31.8	10.1	-17.7	4	-1.4	-0.7
AS-0988	43	Mussle	Mytilus californianus	No	Santa Barbara	7.5	28.1	7.7	-18.9	4.4		
AS-0989	43	Mussle	Mytilus californianus	Yes	Santa Barbara	12.8	51.4	10.7	-18.6	4.7	-3	-0.3

## 2 Experimental Design, Materials and Methods

The goal of this project was to compile a baseline database of δ^13^C and δ^15^N values of plant and animal species that may have been consumed by ancient human populations across southern California in order to further our ability to reconstruct dietary patterns and environmental changes over time. Previous baseline isotope studies from the region mainly targeted marine and terrestrial mammals and terrestrial plants [1], [2], [3], making the expansion of coverage of marine food resources a major focus for this study. We analyzed 80 samples collected from across southern California for this project (see Supplemental Data). A total of 50 different species are represented in the dataset, including 31 plant species, 13 finfish species, 3 mollusk species, 2 crustacean species, and 1 echinoderm. When collecting samples for this study we primarily targeted foods that were mentioned as being consumed by indigenous populations in the ethnohistoric record for the region [5]. The collection of all samples took place between 2014 and 2017 and was carried out by the authors of this paper as well as an undergraduate research assistant.

We collected and analyzed 40 plant samples representing 31 different species for this project. Samples were collected from locations across both San Diego and Santa Barbara counties in southern California. Areas targeted included northern San Diego County, as well as the Mission Canyon area of Santa Barbara and trails along the Camino Cielo north of Santa Barbara. Samples were often collected during hikes by the authors or the undergraduate assistant in the aforementioned areas. All plant sample collection occurred during 2015 and 2016 and took place throughout the year. Our Western Sea Purslane (*Sesuvium verrucosum*) sample, was grown from seed at the Biology Field Station at UCSD. The experimental garden at the University of California, San Diego (UCSD) Biology Field Station using local soils and captured rainwater in order to reproduce a natural environment.

When selecting plant samples for analysis we attempted to target plants that are documented as having been used by indigenous groups in southern California. Out of the 31 plant species found in our database, 26 were consumed as food or as medicine by indigenous southern Californian groups [5]. This includes major food resources such as red maids (*Calandrinia ciliate*), blue dicks (*Dichelostemma capitatum*), toyon berry (*Heteromeles arbitifolia*), holly-leaved cherry seeds (*Prunus ilicifolia*), and acorns (*Quercus* sp.). Other plants included in this study were consumed as tea for medicinal purposes. Plants consumed as teas for medicinal purposes include yerba mansa (*Anemopsis californica*) California croton (*Croton californicus*), longstem buckwheat (Erigonum elongatum). California wild rose (*Rosa californica*), and white sage (*Salvia alpina*). Milkweed (*Asclepias* sp.) was not consumed intentionally but was used as a chewing gum. Four species (*Epilobium canum, Keckiella cordifolia, Matteuccia struthiopteris,* and *Sequoia sempervirens*) were not consumed but were instead used for construction or for topical medical purposes but are nonetheless included in our database.

In addition to plant resources, we analyzed a total of 40 marine animal samples representing 19 different species. All finfish samples were taken from wild specimens caught in the Pacific Ocean off the shore of southern California during June of 2016. Most of these samples were purchased from local fishers at the Dockside Harbor Market in San Diego or at Saturday morning Santa Barbara Harbor fish market. These samples were purchased for this project and would have otherwise been sold commercially. One sardine (*Sardinops sagax*) was donated to the authors by local recreational fisher. Crustacean and echinoderm samples were also purchased from local fishers at the Santa Barbara harbor market. Mussel samples (*Mytilus californianus*) were collected in Santa Barbara County by the authors, while the turban snail (*Megastraea undosa*) sample was also purchased at the Santa Barbara Harbor market. Because no animal subjects were alive when acquired for research, no care or use of live animals were preformed and the research is thus not subject to the National Institutes of Health guide for the care and use of Laboratory animals (NIH Publications No. 8023, revised 1978).

Processing of field samples for stable isotope analysis was conducted at the Paleodiet Laboratory at the University of California, San Diego. Laboratory work was conducted by the authors as well as by undergraduate research assistants. Plant samples were placed in glass beakers with ∼80 mL of double distilled water and cleaned in an ultrasonic bath for 15 min. Plant samples were then shredded in a blender (MagicBullet by NutriBullet), dried between 24 and 48 h at 60 °C in a laboratory oven, and then reduced to powder by grinding in an agate mortar and pestle. Powdered plant samples were then passed through a fine sieve (U.S. 100, 149 microns) to homogenize particle sizes. Samples of finfish and invertebrate flesh were removed from the organism with a stainless steel scalpel and cut into small pieces (∼2, 3 mm diam.). Flesh samples were then divided into two aliquots. Because lipids are more depleted in ^13^C than other tissues [6], one aliquot was subjected to a lipid extraction procedure and the other was left untreated. The first aliquot received a 2:1 chloroform: methanol (v/v) treatment for 20 min in an ultrasonic bath to remove lipids, followed by a 10 min ultrasonic bath in double distilled water to remove any remaining adhering lipids. The second aliquot received no treatment. Both aliquots were subsequently frozen and then lyophilized using a Labconco benchtop (50 L) freeze dryer set at –50 °C and 0.133 mb for 24 h.

Stable isotope analyses were conducted in the Analytical Facility at the Scripps Institution of Oceanography, under the direction of Dr. Bruce Deck. For each sample, 1 mg of powdered plant or lyophilized faunal tissue was weighed out and placed into a tin capsule for combustion. Isotopic analyses for δ^13^C and δ^15^N values were accomplished with a Costech 4010 Elemental Analyzer coupled to a Thermo-Finnigan Delta XP Plus mass spectrometer. Stable carbon and nitrogen isotope values were calibrated to the VPDB and AIR international scales, respectively, through a NBS-18 and NBS-19 calibrated internal acetanilide standard (SIO-AN: *δ*^13^C = −29.3‰, *δ*^15^N = +1.2‰). Accuracy and precision of stable isotope measurements were determined through repeated analyses (every 8 samples) of a glycine standard (expected: *δ*^13^C −35.9‰, *δ*^15^N = +11.8‰).The glycine standard was not used in the calibration process. Results of the glycine standard found that *δ*^13^C = −36.0 ± 0.2‰, *δ*^15^N = +11.8 ± 0.2‰. Pooled standard deviations of plant and faunal samples analyzed in duplicate over the period of analysis were documented as *δ*^13^C = ± 0.1‰, and *δ*^15^N = ± 0.1‰.

Measured δ^13^C and δ^15^N values for each of our samples are reported in the Supplemental Data included with this paper. Also included in the Supplemental Data are corrected δ^13^C values to account for the Suess effect as well as for differences between biological tissue types. The Suess effect refers to modern depletion of δ^13^C values in atmospheric CO_2_ caused by the burning of fossil fuels. All modern plant and animal δ^13^C values were corrected to the preindustrial mean using compiled historical records of atmostpheric δ^13^C values [7]. This correction varied between modern samples collected for the present study and hare samples collected in the 1960s and early 1970s. Differential impacts of the Suess effect on δ^13^C values in marine versus terrestrial environments are accounted for by multiplying the Suess effect correction by 0.65 [8,9]. As δ^13^C_collagen_ values are approximately 1.0‰ higher than δ^13^C_muscle_ values [10,11] we subtracted this amount from terrestrial mammal and otter bone collagen values. For cetaceans and pinnipeds, however, we assumed that a major portion of consumed tissues would have been comprised of blubber, which is depleted in ^13^C relative to flesh. For these species we therefore subtracted 5.6‰ from bone collagen δ^13^C values following previous work by Newsome et al. [10]. We assumed muscle and bone collagen δ^15^N values to be equivalent [9].

In addition to the plant and animal samples analyzed as part of this study, we supplement our baseline isotope database with additional data from previously analyzed plant, terrestrial mammals, marine mammals, and invertebrate samples (Supplemental Data). This included the stable isotope values from 36 modern terrestrial plants [1], and nine terrestrial mammals, including two modern hares (*Lepus* spp.) [3] and seven archaeological deer (*Odocoileus hemionus*) [1]. Supplemental marine specimens include seven invertebrates [2], and 24 archaeological marine mammals [1]. A comparison of stable isotope values, the percent C and N values, and the C:N ratios for defatted and untreated faunal specimens can be found in Table 1. Together these data represent the largest currently available baseline stable isotope dataset for southern California. Interpretations from the application of this new baseline database to human isotope datasets using Bayesian mixing models can be found in the research article co-submitted with this data paper [4].

## Ethics Statement

This work did not involve the use of human subjects or data collected from social media platforms. The project did not involve any *in vivo* or live animal experiments and was therefore carried out in accordance with the National Institutes of Health guide for the care and use of Laboratory animals (NIH Publications No. 8023, revised 1978) as well as the ARRIVE guidelines for reporting of *in vivo* experiments.

## CRediT authorship contribution statement

**Mikael Fauvelle:** Conceptualization, Data curation, Formal analysis, Funding acquisition, Investigation, Writing – original draft. **Andrew D. Somerville:** Conceptualization, Funding acquisition, Investigation, Methodology, Writing – review & editing.

## Declaration of Competing Interest

The authors declare that they have no known competing financial interests or personal relationships which have or could be perceived to have influenced the work reported in this article.

## Data Availability

Surf and Turf: A Dataset of Stable Isotope Values of Plants and Animals from Southern California (Original data) (Mendeley Data)

## Appendix Supplementary materials

Image, application 1
